# Carmine for Gastro-Enteric Diagnosis

**Published:** 1914-08

**Authors:** 


**Carmine for Gastro-Enteric Diagnosis.** Seymour Basch, New York, Archiv für Verdauungskrankheiten, No. 1, 1914. The method is harmless and simple. For the demarcation of faeces, it has been used since Adolf Schmidt's publication in 1898. It affords an approximate method for determining the passage of contents through the entire alimentary canal or absolute obstipation, as in a case of colonic cancer cited. It also may be used to determine gastric stagnation or stagnation in an oesophageal dilation, the presence of a filtulous communication, etc. As one of the special uses of the test is cited a woman who had a duodenal sound in place and who believed she had accidentally pulled it up into the stomach, also that her stomach retained the contents too long. Passing a carmine solution through the duodenal tube, and using a stomach tube eight minutes afterward, carmine was shown to be absent.

